# Cardioprotective Effects of Essential Oil of *Lavandula angustifolia* on Isoproterenol-induced Acute Myocardial Infarction in Rat

**Published:** 2015

**Authors:** Mojtaba Ziaee, Arash Khorrami, Maryam Ebrahimi, Hassan Nourafcan, Masoumeh Amiraslanzadeh, Maryam Rameshrad, Mehraveh Garjani, Alireza Garjani

**Affiliations:** a *Department of Pharmacology, Miyaneh Branch, Islamic Azad University, Miyaneh, Iran. *; b *Department of Pharmacology and Toxicology, Faculty of Pharmacy, Tabriz University of Medical Sciences, Tabriz, Iran. *; c *Student Research Committee; Tabriz University of Medical Sciences, Tabriz, Iran.*

**Keywords:** Myocardial infarction, *L. angustifolia*, Antioxidant, Isoproterenol

## Abstract

Myocardial infarction (MI) is a common presentation of the ischemic heart disease. *Lavandula angustifolia* is an herbaceous plant with antioxidative effects. This study was designed to investigate the cardioprotective effects of *lavandula angustifolia *essential oil against isoproterenol-induced MI in rats. The dried sample was subjected to hydrodistillation by using a Clevenger and the oils were dried over anhydrous Na_2_SO_4_. Male Wistar rats were assigned to 6 groups of control, sham, isoproterenol and treatment with 5, 10, 20 mg/Kg of the essential oil. MI was induced by subcutaneous injection of Isoproterenol (100 mg/Kg) for 3 consecutive days at an interval of 24 h. The essential oil was given intraperitoneally every 24 h started at MI induction. Following anesthesia, hemodynamic parameters were measured. After sacrificing the animals, the hearts were removed to measure the heart to body weight ratio and histopathological examination. Myeloperoxidase (MPO) and Malondialdehyde (MDA) were measured in heart tissues for evaluating the activity of neutrophils and lipid peroxidation, respectively. The essential oil amended ECG pattern by suppressing ST-segment elevation and increasing R-amplitude. 10 mg/Kg of the essential oil significantly decreased heart to body weight ratio (P<0.001) and the elevation of MDA and MPO in myocardium, it also increased dp/dtmax from 2793 ± 210 to 4488 ± 253 mmHg/sec (P<0.001), and 20 mg/Kg of it significantly lowered LVEDP from 14 ± 3.43 to 4.3 ± 0.83 mmHg (P<0.001).The results demonstrated that *L. angustifolia* protects myocardium against isoproterenol-induced MI that it could be related to its antioxidant properties.

## Introduction

*Lavandula angustifolia *(Lamiaceae) known in Iran as “ Ostokhoddus “ is a strongly aromatic shrub with 1-2 m height and is a commonly used household herb(1,2). It is native to the Mediterranean regions and is commercially cultivated in France, Spain, Portugal, UK, Bulgaria, Australia, China, and the USA. Lavender oil is famous due to its delightful aroma and has extensive application in the perfumery, colognes, skin lotions and other cosmetic industries (3).

Lavander oil which is prepared from the flowers of *L. angustifolia *(1.5-3%), is consist of linalyl acetate (25-55%), linalool (20-30%), 1,8-cineol (0.3-1.5%), lavandulol, lavandulyl acetate, camphor (0.2-0.5%), cis-β-ocimene (4-10%), trans-β-ocimene (2-6%), 1-terpinen-4-ol (2-6%), α-terpineol (0.3-1%), limonene (0.1-0.5%), tannins (5-10%), coumarins, flavonoids (luteolin), phytosterols and triterpens. lavender essential oil is used in food manufacturing as an additive flavor in beverages, ice-cream, candy, baked goods and chewing gum (4).

In Iranian folk and traditional medicine *L. angustifolia* has been used as carminative, diuretic, antiepileptic, anti-rheumatic and pain reliever especially in nervous headache and migraine. In pharmacological and biological tests, extracts, fractions, and essential oil of *L. angustifolia* demonstrated therapeutic effects such as CNS-depressant, anticonvulsive, antibacterial and mast cell degranulation inhibitory effects (1).

Several medicinal properties and biological activities have been proved for *L. angustifolia* essential oil are listed here: anti-inflammatory (3), antioxidant (4), antiseptic, antispasmodic, carminative, diuretic, sedative (5), expectorant, mucolytic (6), antiflatulence, anti-colic (7).

Acute myocardial infarction is an important ischemic heart disease and it is a leading cause of morbidity and mortality worldwide. Isoproterenol is a synthetic β-adernoceptor agonist that its subcutaneous injection results in myocardial infarction in rats (8), which causes irreversible cellular damage and ultimately infarct-like necrosis (9, 10). The acute phase of myocardial necrosis induced by isoproterenol is defined by changes in blood pressure, heart rate, electrocardiogram (ECG), and left ventricular dysfunction similar to that occurs in patients with myocardial infarction. The rat model of isoproterenol-induced myocardial infarction is a reliable non-invasive method for evaluating the effects of various potential cardioprotective agents (11). Among different possible mechanisms offered to explain isoproterenol induced cardiac damage, generation of highly cytotoxic free radicals through auto oxidation of catecholamines is reported to be one of the important causative factors (12). Although *L. angustifolia*, as an important medicinal plant, shows strong antioxidant properties (1,4,21), its cardioprotective activity against isoproterenol induced myocardial infarction has not been elucidated. The aim of the present study is to investigate the effect of Intraperitoneal (IP) administration of essential oil of aerial parts of *L. angustifolia* on isoproterenol induced myocardial injury. Left ventricular dysfunction, oxidative stress, and histopathological changes induced by isoproterenol were examined and their modulation with different doses of *L. angustifolia* essential oil was evaluated.

## Experimental

*Plant material*

The aerial parts of *Lavandula angustifolia *were collected during flowering stage from Miyaneh, Azarbaijan shargi-Iran, and then were dried. 

*Essential oil preparation*

 The dried aerial parts of the plant (100 g) were subjected to hydrodistillation by using a Clevenger apparatus for 3 h. The oils were dried over anhydrous Na_2_SO_4_ and stored in seal vials at 4 ^0^Ċ. 

*Animals*

Male albino Wistar rats (260-280 g) were used in this study. Rats were housed at constant temperature (20 ± 1.8 ^0^Ċ) and relative humidity (50 ± 10%) in standard polypropylene cages, six per cage, under a 12 h light/dark. They were fed with commercial rat cube diet and given water *ad libitum*. This study was performed in accordance with the guide for the care and use of laboratory animals of Tabriz University of medical sciences, Tabriz-Iran (National institutes of health publication No 85-23 revised 1985).

*Induction of acute myocardial infarction*

Isoproterenol was dissolved in normal saline and injected subcutaneously (S.C) to rats (100 mg/Kg) for 3 consecutive days at an interval of 24 h to induce acute myocardial infarction. Animals were sacrificed 72 h after the first dose of isoproterenol.

*Experimental protocol*

The animals were randomly allocated into 6 groups. Sample size of each group was 6 rats. Rats in group 1 (normal control) received a S.C injection of normal saline (0.5 mL) and were left untreated for the whole period of the experiment. In group 2 (Sham) rats received a S.C injection of normal saline and *L. angustifolia* essential oil was injected intraperitoneally (IP) (20 mg/Kg) for 3 days. Rats in group 3 (Isoproterenol group) received a S.C injection of isoproterenol (100 mg/Kg) for 3 consecutive days at an interval of 24 h. Concurrently with induction of MI, rats in group 4-6 were injected intraperitoneally (IP) essential oil, dispersed in saline, at doses of 5, 10, 20 mg/Kg, for 2 days.

*Hemodynamic measurements*

 Animals were anaesthetized by intraperitoneal (IP) injection of ketamine (0.5 mL), xylazine (0.3 mL) and acepromazine (0.2 mL) mixture 72 h after the first dose of isoproterenol. The trachea was cannulated and the animals were allowed to breathe spontaneously. The systemic arterial blood pressure (SABP) was recorded from a catheter inserted to the left carotid artery. A standard limb lead II ECG was monitored continuously throughout the experimental period. The mean arterial blood pressure (ABP) was calculated from the systolic and diastolic blood pressure traces. The heart rate was calculated from ECG. To evaluate the cardiac left ventricular function, a Mikro Tip catheter transducer (Millar Instruments.INC) was passed via the right carotid and down into the left ventricle. This was used to measure the left ventricular systolic pressure (LVSP), left ventricular end-diastolic pressure (LVEDP), maximum and minimum rates of rise of the left ventricular pressure (Lvdp/dtmax and Lvdp/dtmin) (indices of contractility), and to measure the rate of pressure change at a fixed left ventricular pressure (Lvdp/dt/p) (In this case 50 mmHg). All the parameters were continuously recorded using a power lab system (AD Instrument. Australlia). The calibration of the transducer was checked daily against a mercury column.

*Tissue weights*

 After the hemodynamic measurement, the animals were killed by an overdose of an anesthetic and the hearts were removed and weighted. The heart weight to body weight ratio was calculated to assess the degree of congestion.

*Histopathological examinations*

To assess the heart remodeling , apex part of the hearts were embedded in 10% buffered formalin, then the tissues were fixed in paraffin, sectioned in 5μm thickness and stained with hematoxylin and eosin (H&E) for evaluation of histology and neutrophil infiltration and edema. Two persons (one-blind form) graded the histopathological changes as 1, 2, 3 and 4 for low, moderate, high and intensive pathological changes, respectively.

*Determination of lipid peroxidation in myocardium*

 Malondialdehyde (MDA), a thiobarbiturate reactive substance was measured as a marker for oxidative stress in myocardial homogenates. A standard curve was prepared for TBA method for the calculation of the tissue MDA using 1, 1, 3, 3-tetraethoxypropan (TEP) (1.25, 2.5, 5, 10, 20 nmol/mL) (y=55.7x+0.7; r=0.994). The lipid peroxide was expressed as nanomole MDA production per gram heart tissue. To determine the level of MDA in the tissues, the hearts were homogenized in a ratio of 1/10 in 1.15% (w/v) cold KCl solutions. Then 0.5 mL of the heart homogenate was shaken with 3 mL of 1% phosphoric acid in a 10 mL centrifuge tube. 1ml of 0.6% TBA was added to the mixture, shaken, and warmed for 45min in a boiling water bath. After cooling, 4 mL of *n*-butanol was added to the tubes and mixed vigorously. Then tubes were centrifuged for 15 min at 5000 rpm and MDA content in the serum was determined from the absorbance at 535 by spectrophotometer against *n*-butanol (13).

*Myeloperoxidase assay*

 Myeloperoxidase (MPO) was measured in tissue for quantifying the activity of the neutrophils in myocardium (14). The tissue samples were homogenized (IKA Homogenizer, Staufen ,Germany) in a solution containing 0.5% hexa-decyltrimethyl-ammonium bromide (HTAB) dissolved in 50 mM potassium phosphate buffer(PH=6). The samples were then centrifuged at 4500 rpm for 20 min at 4 ^0^Ċ. The samples were freeze-thawed three times and sonicated for 20 s. An aliquot of the supernatant (0.1 mL) or standard (Sigma, Germany) was then allowed to react with 2.9 mL solution of 50mM potassium phosphate buffer at PH=6 containing 0.167 mg/mL of o-dianisidine hydrochloride and 0.0005% H_2_O_2_. After 5 min the reaction was stopped with 0.1 mL of 1.2 M hydrochloric acid. The rate of change in absorbance was measured using a spectrophotometer (Cecil 9000, Cambridge UK) at 460 nm. A standard curve using standard myeloperoxidase (1500-25000 mU/g) (Sigma, Germany) was drawn to determine the activity of the enzyme. Myeloperoxidase activity was expressed in milli unit (mU) per gram weight of the wet tissue.

*Statistics*

 Data were presented as mean ± SEM. One-way-ANOVA was used to make comparisons between the groups. If the ANOVA analysis indicated significant differences, a Student–Newman–Keuls post test was performed to compare the mean values between the treatment groups and the control. Any differences between groups were considered significant at P<0.05.

## Results

*The effects of L. angustifolia essential oil on electrocardiographic pattern and parameters*

**Figure 1:**
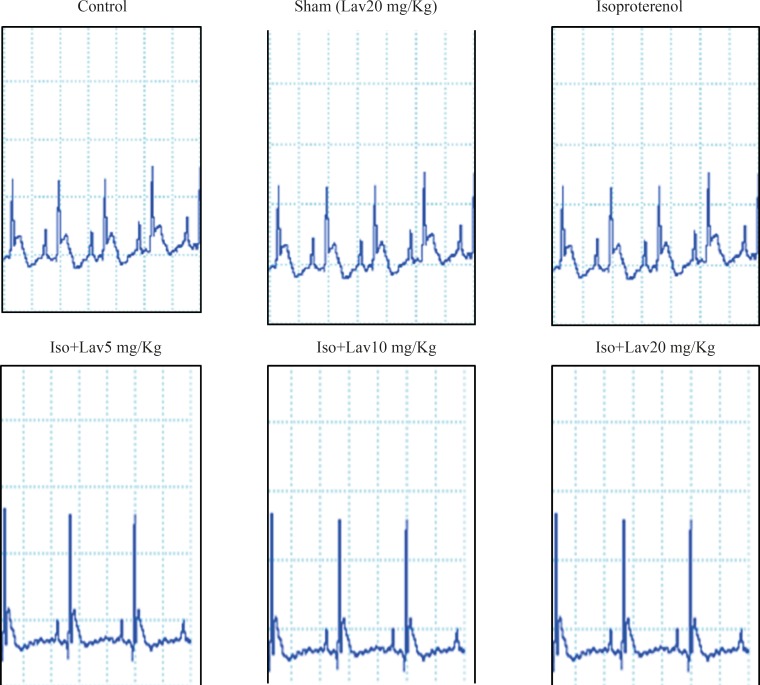
The effects of essential oil of *L. angustifolia* on electrocardiographic pattern and changes (recorded from limb lead II) in control, isoproterenol and Lavandula groups. Iso: Isoproterenol, Lav: *L. angustifolia* essential oil.

**Figure 2:**
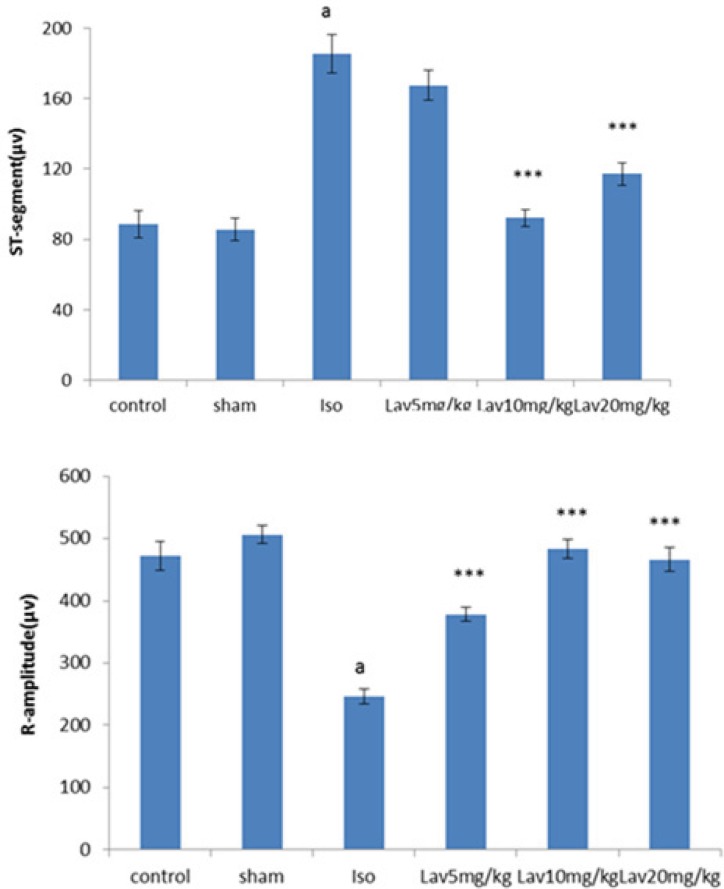
Effects of the essential oil of *L. angustifolia* on ST-segment height and R-amplitude. Values are mean ± SEM (n=6). ᵃP<0.001 from respective control value, ***P<0.001 as compared with isoproterenol treated group using one way ANOVA with Student-Newman-Keuls post hoc test. Iso: Isoproterenol, Lav: *L. angustifolia* essential oil.

The normal control group and the rats that had received the essential oil alone (20 mg/Kg, sham) showed normal patterns of ECG, whereas the rats treated with isoproterenol, exhibited a marked (P<0.001) reduction in R-amplitude along with a significant (P<0.001) elevation of ST-segment, indicative of myocardial infarction. (Figure 1). Treatment with 10 and 20 mg/Kg of essential oil demonstrated a profound reduction (P<0.001) in the ST-segment elevation and treatment with all doses of essential oil resulted in a marked (P<0.001) increase in the R-amplitude as compared to ECGs obtained from isoproterenol alone treated rats (Figure 2).

*The effects of essential oil of L. angustifolia on hemodynamic parameters*

The mean arterial blood pressure (MAP) was significantly decreased from 80 ± 6.04 mmHg in normal control to 36.5 ± 3 mmHg in the isoproterenol treated group (P<0.001). MAP was increased significantly to 63 ± 4.5, 80 ± 3.08 and 67 ± 4.76 mmHg in treated groups with 5, 10 and 20 mg/Kg of *L. angustifolia* essential oil, respectively (P<0.001, Table 1).

The heart rate (HR) was significantly increased from 193.75 ± 7.11 (bpm) in control group to 296 ± 20.62 (bpm) in isoproterenol treated group (P<0.001). There was a significant (P<0.01) decrease in HR to 202 ± 9.1 and 203 ± 15.5 (bpm) respectively after treatment with 10 and 20 mg/Kg *L. angustifolia* essential oil (Table1).

The intraventricular pressure was measured to determine the degree of left ventricular responses to the isoproterenol injection. Isoproterenol significantly reduced the left ventricular systolic pressure (LVSP) from 114 ± 6.8 in normal control group to 75 ± 2.8 mmHg (P<0.001). Lavandula essential oil at 10 mg/Kg caused a significant increase in LVSP to 115 ± 6 mmHg (P<0.001, Table1).

There was almost a 3fold elevation in the left ventricular end diastolic pressure (LVEDP) of isoproterenol alone treated rats, thereby indicating left ventricular dysfunction. All three doses of the essential oil considerably (P<0.01, P<0.001) improved the left ventricular function by lowering LVEDP from 14 ± 3.43 in the rats with myocardial infarction to 5.7 ± 1.43, 5.2 ± 0.62 and 4.3 ± 0.85 mmHg respectively (Table1).

**Figure 3:**
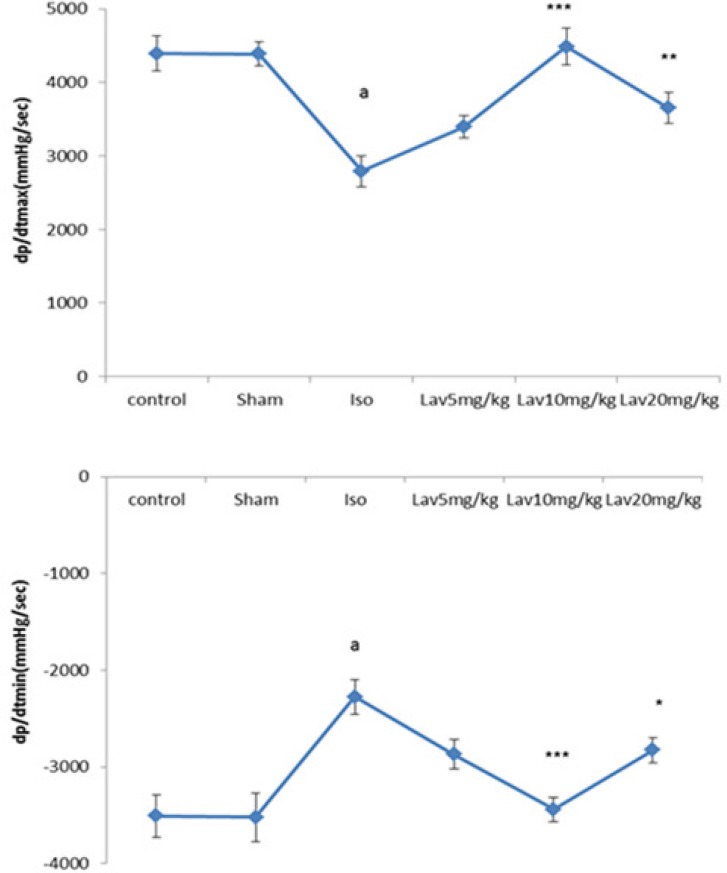
Left ventricular maximal and minimal rates of pressure increase (LVdp/dtmax, LVdp/dtmin) in the control group and in the rats treated with isoproterenol alone (rats with myocardial infarction), *L. angustifolia* alone (Sham), and Isoproterenol plus *L. angustifolia*. Values are mean±SEM (n=6). ªp<0.001 , ᵇp<0.01 ,ᶜp<0.05 from respective control value; *p < 0.05, **p < 0.01; ***p < 0.001 as compared with isoproterenol treated group using one way ANOVA with Student-Newman-Keuls post hoc test.

When compared with the normal control, the rats with left ventricular dysfunction (isoproterenol group) demonstrated a fall in the values of the left ventricular maximal and minimal rates of pressure (LVdp/dtmax, LVdp/dtmin. P<0.001. Figure 3) as well as lower rate of pressure change at a fixed ventricular pressure (LVdp/dt/p, P<0.001, Table1). LVdp/dtmax (indice of myocardial contractility) increased significantly in treated rats with 10 and 20 mg/Kg of essential oil, P<0.001 and P<0.01. Figure 3). LVdp/dtmin also showed a marked improvement (P<0.001, P<0.05) by Lav10 and 20 mg/Kg, respectively.

**Table1:** The effects of essential oil of *L. angustifolia* on hemodynamic parameters.

**Groups**	**MAP** **(mmHg)**	**Herat rate** **(bpm)**	**LVSP ** **(mmHg)**	**LVEDP** ** (mmHg)**	**LV dP/dt/P ** **(1/sec)**
N=6					
Control Sham Isoproterenol *L. angustifolia *(5 mg/Kg) + isoproterenol *L. angustifolia*(10 mg/Kg) + isoproterenol *L. angustifolia *(20 mg/Kg) + isoproterenol	80±6.04 80±3.96 36±2.98ᵃ 63±4.4 *** 80±3.08 *** 67±4.76 ***	193±7 204±12 269±20.62ᵃ 242±15 202±9 ** 203±15.5**	114±6.8 112±7.3 75±2.8ᵃ 85±4.9 115±6 *** 91±3.6	5.8±0.31 7.5±0.95 14.3±3.4 ᵇ 5.7±1.4 ** 5.2±0.6 ** 4.3±0.85 ***	85±3.9 85±3.6 54±2.5ᵃ 68±1.4 * 86±3.7 *** 70±4.1 **

ª p<0.001 ,

ᵇ p<0.01 ,

ᶜ p<0.05 from respective control value;

* p < 0.05,

** p < 0.01;

*** p < 0.001 as compared with isoproterenol treated group using one way ANOVA with Student-Newman-Keuls post hoc test.

**Figure 4:**
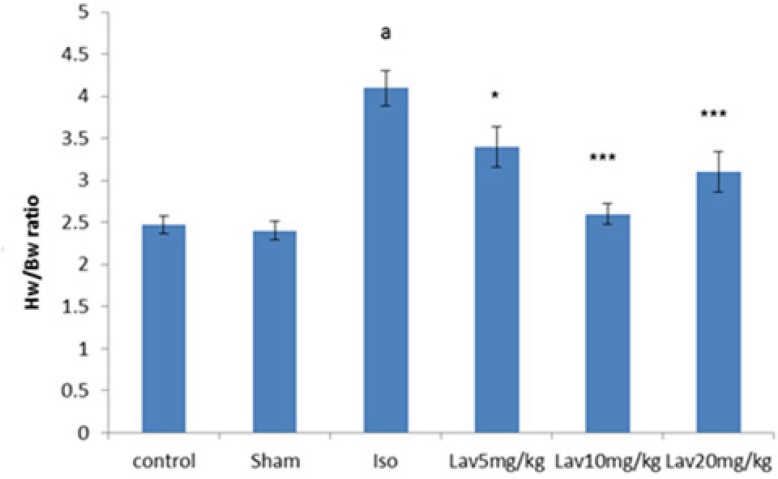
The effects of *L. angustifolia* essential oil on Heart weight (HW) to Body weight (BW) ratios. Data are expressed as mean±SEM (n=6). ªp<0.001 , ᵇp<0.01 ,ᶜp<0.05 from respective control value; *p < 0.05, **p < 0.01; ***p < 0.001 as compared with isoproterenol treated group using one way ANOVA with Student-Newman-Keuls post hoc test. Iso: Isoproterenol, Lav: *L. angustifolia* essential oil.

In order to assess the extent of heart weight gain developed by injection of isoproterenol, the heart weight to body weight ratio (HW/BW) was determined (Figure 4). The ratio was significantly (P<0.001) in the isoproterenol treated rats (4.1 ± 0.21) compared with the normal control group (2.47 ± 0.1). Intraperitoneal (IP) treatment with all doses of *L. angustifolia* essential oil produced reduction in the heart weight to body weight ratio. (P<0.05, P<0.001 and P<0.001, respectively, Figure 4).

*Histopathological examination of the cardiac tissues*

**Figure 5:**
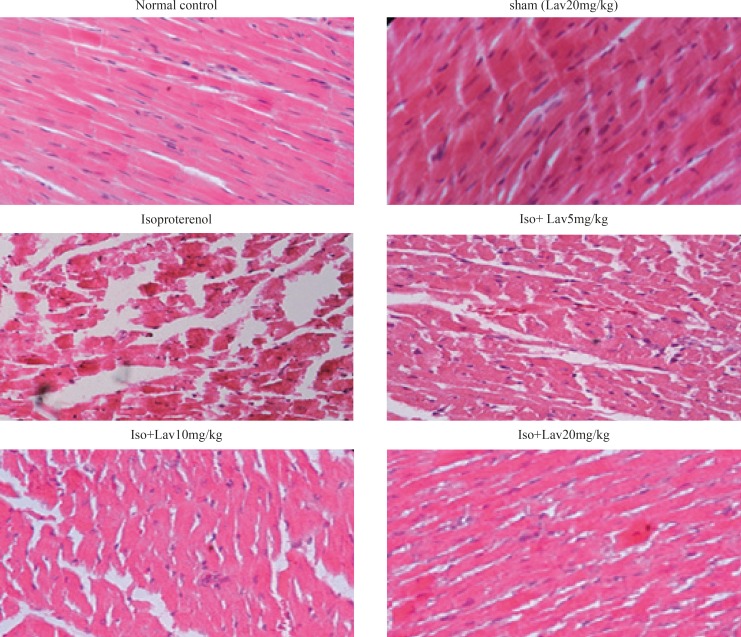
Photomicrographs of sections of rat cardiac apexes. Heart tissue of a rat subcutaneously injected with isoproterenol alone shows intensive cardiomyocyte necrosis and increased edematous intramuscular space. Treatment with *L. angustifolia* demonstrates a marked improvement. Iso: Isoproterenol. Lav: *L. angustifolia* (H&E).

**Figure 6:**
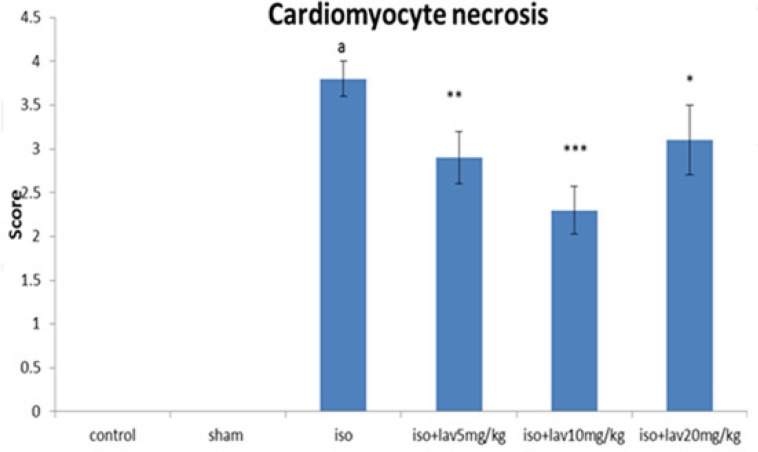
The effects of *L. angustifolia* administration on grading of histopathological changes in rats’s cardiac apex tissues (scores 1, 2, 3, and 4 show low, moderate, high and intensive pathological changes respectively). Data are expressed as mean ± sem (n =6). ªp <0.001 from respective control value; *p < 0.05; **p < 0.01; ***p < 0.001 as compared with isoproterenol treated group using one way ANOVA with Student-Newman-Keuls post hoc test. Iso: Isoproterenol, Lav: *L. angustifolia* essential oil.

In the normal control group, myocardial fibers were arranged regularly with clear striations. No apparent degeneration or necrosis was observed (Figure 5). Histological sections of the isoproterenol treated hearts showed widespread subendocardial necrosis, hypertrophy, and abundant fibroblastic hyperplasia along with increased edematous intramuscular space. *L. angustifolia* treatment to isoproterenol groups showed a significant protection against myocardial injury. The *L. angustifolia* essential oil with doses of 5, 10, and 20 mg/Kg reduced the isoproterenol-induced necrosis and edematous as shown in (Figure 6). 

*Effects of essential oil of L. angustifolia on Malondialdehyde (MDA) level*

**Figure 7:**
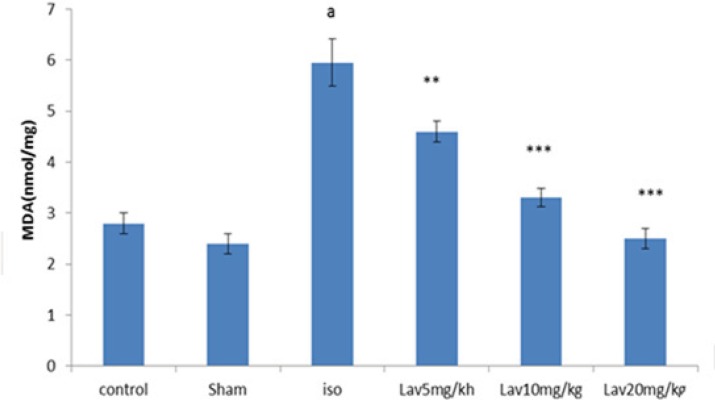
The effects of *L. angustifolia* essential oil on malondialdehyde (MDA). Data are expressed as mean±SEM (n=6). ªp<0.001 , ᵇp<0.01 ,ᶜp<0.05 from respective control value; *p < 0.05, **p < 0.01; ***p < 0.001 as compared with isoproterenol treated group using one way ANOVA with Student-Newman-Keuls post hoc test. Iso: Isoproterenol, Lav: *L. angustifolia* essential oil.

To determine the lipid peroxidation, MDA level was measured in myocardial homogenates. MDA level was considerably increased (P<0.001) in isoproterenol injected rats (MI group) in comparison with normal control. Treatment with all doses of essential oil markedly diminished the myocardial MDA (P<0.01, P<0.001, P<0.001 respectively. Figure 7).

*Effects of essential oil of L. angustifolia on Myeloperoxidase (MPO) assay*

**Figure 8:**
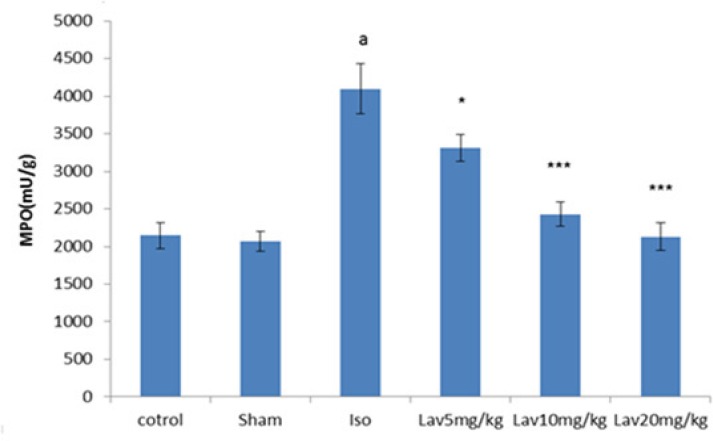
The effects of *L. angustifolia* essential oil on myeloperoxidase (MPO). Data are expressed as mean±SEM (n=6). ªp<0.001 , ᵇp<0.01 ,ᶜp<0.05 from respective control value; *p < 0.05, **p < 0.01; ***p < 0.001 as compared with isoproterenol treated group using one way ANOVA with Student-Newman-Keuls post hoc test. Iso: Isoproterenol, Lav: *L. angustifolia* essential oil.

To quantify the activity of neutrophils in myocardium, MPO was measured in myocardial homogenates. MPO was significantly (P<0.001) increased in isoproterenol treated rats (MI group) in comparison with normal control. Treatment with all doses of essential oil markedly decreased the myocardial MPO (P<0.05, P<0.001, P<0.001, respectively. Figure 8).

## Discussion

Myocardial infarction (MI) commonly known as heart attack, is an acute condition of necrosis of myocardium is essentially developed as a result of imbalance between oxygen need and actual supply (15). Lavander essential oil is believed to have efficacy against variety of problems including stress, anxiety, exhaustion, irritability, insomnia, depression, colds, digestion, flatulence, upset stomach, liver and gallbladder problems, nervousness, loss of appetite (16). However, little is known about its cardioprotective actions in cardiovascular diseases. In the present study, the therapeutic efficacy of the essential oil of the aerial parts of the plant was investigated in rats with acute myocardial infarction induced by isoproterenol. The ECG is considered the most important clinical test for diagnosis of myocardial infarction. The ST-segment elevation reflects the potential difference in the boundary between ischemic and non-ischemic zones and the consequent loss of cell membrane function, whereas the decreased R-amplitude might be due to the onset of myocardial edema following isoproterenol administration (17). The animals on *L. angustifolia* treatment showed an obvious improvement in their ECG pattern, indicating its protective effects on cell membrane functions. When administrated for a period of more than 24 h, high-dose β-agonists results in myocardial edematous and histological changes that include myocyte necrosis, myofibrilar degeneration, and leukocyte infiltration (18). Intraperitoneal (IP) injection of the essential oil in doses of 5, 10, and 20 mg/Kg for two days clearly and dose dependently prevented myocardial injury and edematous in the isoproterenol treated rats, indicating its anti-edematogenic activity. Treatment with all doses of essential oil decreased significantly heart to body weight ratio, demonstrating the preventive effect of the essential oil on the cardiac edematous and hypertrophy. These effects could be related to anti-inflammatory and anti-edematous properties of linalool and linalyl acetate which are main components of *L. angustifolia *(22). The subcutaneous injection of isoproterenol also significantly decreased the arterial pressure indices, left ventricular contractility (LVdP/dtmax) and relaxation (LVdP/dtmin), and increased the left ventricular end-diastolic pressure (LVEDP). In the treatment of post myocardial infarction heart failure it is very important to improve the contractility of the heart and at the same time decrease the left ventricular end diastolic pressure. In the present study, the beneficial effects of the *L. angustifolia* essential oil were clearly observed with respect to a variety of indices including improvement of arterial blood pressure, increase in myocardial contractility force, and parallel decrease in the left ventricular end diastolic pressure. Generation of reactive oxygen species (ROS) lead to the progression of ventricular remodeling after myocardial infarction because of initiation of peroxidation of membrane bound polyunsaturated fatty acids (PUFAs) (19,20). In addition to *in-vitro* antioxidant activities of essential oil in linoleic acid system (16), the results of the present study demonstrated *in-vivo* antioxidant activity of *L. angustifolia* essential oil, so that the essential oil diminished the myocardial lipid peroxidation by 50-60%. A high dose of essential oil (20 mg/Kg) produced the strongest reduction on the level of MDA in myocardium. In this study, isoproterenol injected animals, revealed a significant increase in myocardial MPO level, demonstrative of necrosis induced inflammation of the heart tissue and neutrophil infiltration into the blood and heart tissue (as site of inflammation). Treatment with all doses of *L. angustifolia *essential oil (5, 10, and 20 mg/Kg), significantly decreased the level of MPO (inflammatory marker). This reduction reflects decreased neutrophil infiltration into the myocardium. The anti-inflammatory effect of essential oil seen in this study, could be attributed to the presence of linalool and linalyl acetate. Myocardial healing was seen with different dose of the essential oil of *L. angustifolia* in isoproterenol treated rats suggesting a potent protective effect of the essential oil on myocardial infarction which could be related to its antioxidant activities. According to phytochemical studies, linalool, linalyl acetate and some other mono and sesquiterpenes, camphor, 1,8-cineol, flavonoids like luteolin, triterpenoids like ursolic are and coumarin are the main constituents of the essential oil (5), so we can conclude that most of the therapeutic effects of *L. angustifolia* essential oil might be due to these components.

## Conclusion

 The essential oil of *L. angustifolia* exerts cardiprotective effects probably because of its anti-inflammatory, free radical scavenging and antioxidant activities. Thus essential oil of *L. angustifolia* protected the myocardium against isoproterenol-induced MI by normalizing ECG, improvement the hemodynamic impairment, decreasing lipid peroxidation, suppressing proinflammatory responses, and improving antioxidant systems. Essential oil of lavandula maintained the structure and architecture of cardiac cells by decreasing cardiac tissue damage and strengthening myocardial membrane as evidenced by histopathological results of myocardium. If the beneficial effects of *L. angustifolia* essential oil could be reproduced in human beings; our findings may introduce a novel therapy for prevention and treatment of myocardial infarction.
